# Obesity and risk for respiratory diseases: a Mendelian randomization study

**DOI:** 10.3389/fendo.2023.1197730

**Published:** 2023-08-29

**Authors:** Wenwen Yang, Yanjiang Yang, Yan Guo, Jinde Guo, Minjie Ma, Biao Han

**Affiliations:** ^1^ The First Clinical Medical College, Lanzhou University, Lanzhou, Gansu, China; ^2^ The people’s Hospital of Qiandongnan Autonomous Prefecture, Kaili, Guizhou, China; ^3^ Dingxi City People’s Hospital, Dingxi, Gansu, China; ^4^ Acupuncture and Massage, Gansu University of Chinese Medicine, Lanzhou, Gansu, China; ^5^ Department of Thoracic Surgery, the First Hospital of Lanzhou University, Lanzhou, Gansu, China; ^6^ Gansu Province International Cooperation Base for Research and Application of Key Technology of Thoracic Surgery, The First Hospital of Lanzhou University, Lanzhou, Gansu, China

**Keywords:** Mendelian randomization, obesity, FinnGen biobank, respiratory diseases, UK Biobank

## Abstract

**Background:**

No existing comprehensive Mendelian randomization studies have focused on how obesity affects respiratory diseases.

**Methods:**

BMI and waist circumference, mainly from the UK Biobank, and 35 respiratory diseases from the FinnGen Biobank were subjected to Mendelian randomization analyses. In this study, the inverse variance weighting method was used as the predominant analysis method and was complemented by MR-Egger and weighted median methods. Horizontal pleiotropy and potential outliers were detected by employing the MR-PRESSO method.

**Results:**

This study indicated that obesity rises the possibility of acute upper respiratory infections (BMI: OR=1.131, p<0.0001; WC: OR=1.097, p=0.00406), acute sinusitis (BMI: OR=1.161, p=0.000262; WC: OR=1.209, p=0.000263), acute pharyngitis (WC: OR=1.238, p=0.0258), acute laryngitis and tracheitis (BMI: OR=1.202, p=0.0288; WC: OR=1.381, p=0.00192), all influenza (BMI: OR=1.243, p=0.000235; WC: OR=1.206, p=0.0119), viral pneumonia (WC: OR=1.446, p=0.000870), all pneumoniae (BMI: OR=1.174, p <0.0001; WC: OR=1.272, p <0.0001), bacterial pneumoniae (BMI: OR=1.183, p=0.000290; WC: OR=1.274, p<0.0001), acute bronchitis (BMI: OR=1.252, p <0.0001; WC: OR=1.237, p=0.000268), acute unspecified lower respiratory infection (BMI: OR=1.303, p=0.000403), chronic tonsils and adenoids diseases (BMI: OR=1.236, p <0.0001; WC: OR=1.178, p=0.000157), chronic laryngotracheitis and laryngitis (WC: OR=1.300, p=0.00785), COPD (BMI: OR=1.429, p <0.0001; WC: OR=1.591, p <0.0001), asthma (BMI: OR=1.358, p <0.0001; WC: OR=1.515, p <0.0001), necrotic and suppurative conditions of lower respiratory tract (WC: OR=1.405, p=0.0427), pleural effusion (BMI: OR=1.277, p=0.00225; WC: OR=1.561, p<0.0001), pleural plaque (BMI: OR=1.245, p=0.0312), other diseases of the respiratory system (BMI: OR=1.448, p <0.0001; WC: OR=1.590, p <0.0001), and non-small cell lung cancer (BMI: OR=1.262, p=0.00576; WC: OR=1.398, p=0.00181). This study also indicated that obesity decreases the possibility of bronchiectasis (BMI: OR=0.705; p=0.00200).

**Conclusion:**

This study revealed that obesity increases the risk of the majority of respiratory diseases (including 20 of all 35 respiratory diseases) and that obesity decreases the risk of bronchiectasis.

## 1 Introduction

Obesity has become a global health issue. There are more than 2 billion overweight individuals worldwide (1). In more than 70 countries, the incidence of obesity has risen by twofold since 1980 and is continually rising (2). Obesity is a chronic condition that can impact practically all body organs and tissues. Obesity and respiratory diseases have been linked in numerous earlier studies (3–16). In 1984, Xanthopoulos M et al. (5) reported a link between overweight and asthmatic respiratory symptoms. A large number of subsequent studies have explored the relationship between body mass index (BMI) and asthma (6–16). The majority of these studies, all but three (10, 11, 15), found that obese people had higher incidences of asthma. Meta-analyses have found that an increase in BMI is associated with an increased risk of pulmonary embolism (17) and a reduced risk of COPD (18). There have previously been several Mendelian randomization (MR) analyses that focus on the association between body mass index and respiratory conditions including asthma (19), COPD (20), pneumonia (21), and chronic rhinosinusitis (22). On the one hand, their study was limited to employing BMI as the only indicator of obesity and did not evaluate the impact of body fat distributions. On the other hand, the association between obesity and the risk of respiratory illness has not been systematically studied. We searched the FinnGen Biobank (round 8) for respiratory diseases according to ICD-10 (Version: 2016) and finally included 35 respiratory diseases. This study employs MR analytical methods to investigate obesity and 35 respiratory disorders, which makes it highly important for a thorough knowledge of how obesity affects respiratory disorders.

## 2 Methods

For MR analysis, the assumptions listed below must be satisfied. First, instrumental variables (IVs) must be closely linked to the exposure factors. Second, there is no direct relationship between IVs and the outcomes. Finally, there was no connection between IVs and any possible confounding factors. The GWAS datasets utilized in this study were released by the FinnGen Biobank and IEU open GWAS project. This open GWAS project, developed at the University of Bristol’s MRC Integrative Epidemiology Unit (IEU), compiled and examined GWAS datasets from the UK Biobank and existing studies (23). The data utilized in this study are deidentified and publicly available, therefore this study was excluded from the Institutional Review Board’s approval requirements.

### 2.1 Sources of data

This study employed waist circumference and body mass index as obesity-related exposures. The GWAS summary-level data for waist circumference and BMI used in this study were extracted from a published article (24) and the UK Biobank. The GWAS summary-level data for 35 respiratory diseases, including 34 ICD-10-based respiratory diseases and one ICD-O-3-based non-small cell lung cancer (NSCLC), were all publicly released in Round 8 of the FinnGen biobank (25). The Disease Code in ICD-10 for these diseases is shown in **Table 1**. We did not employ proxy single-nucleotide polymorphisms (SNPs) while reviewing the outcomes for SNPs that were associated with exposures because the majority of SNPs were discovered in the outcomes. the participants of BMI and waist circumference were 681275 and 462166 European-descent individuals, respectively. The range of European-descent cases in the outcomes is from 1035 to 61543. We selected respiratory diseases strictly according to ICD-10 and we did not exclude any disease unless the number of cases was less than 1000 or the Finngen Biobank did not have data for this disease. **Table 1** provides more details about the exposures and outcomes.

**Table 1:** Information of the exposures and outcome datasets.

IEU id/ Endpoint name in FinnGen Biobank	Exposure or outcome	category	Number of cases	Number of controls	Disease Code in ICD-10
ieu-b-40	body mass index	NA	681275 European-descent individuals		NA
ukb-b-9405	Waist circumference	NA	462166 European-descent individuals		NA
J10_UPPERINFEC	Acute upper respiratory infections	X Diseases of the respiratory system (J10_)	61543	280956	(J00-J06)
J10_COLD	Acute nasopharyngitis(common cold)	X Diseases of the respiratory system (J10_)	3554	280956	J00
J10_SINUSITIS	Acute sinusitis	X Diseases of the respiratory system (J10_)	18317	280956	J01
J10_PHARYNGITIS	Acute pharyngitis	X Diseases of the respiratory system (J10_)	4140	280956	J02
J10_LARYNGITIS	Acute laryngitis and tracheitis	X Diseases of the respiratory system (J10_)	3689	280956	J04
J10_ACUTEUPPERINFEC	Acute upper respiratory infections of multiple and unspecified sites	X Diseases of the respiratory system (J10_)	32319	280956	J06
J10_INFLUENZA	All influenza	X Diseases of the respiratory system (J10_)	7580	286619	J9-11
J10_VIRALPNEUMO	Viral pneumonia	X Diseases of the respiratory system (J10_)	2915	286619	J12|J171|J100|J110|B012|B068|B250
J10_PNEUMONIA	All pneumoniae	X Diseases of the respiratory system (J10_)	52021	290478	J12 J13 J14 J15, J17.0*, J17.0*A01.0, J17.0*A02.2, J17.0*A21.2, J17.0*A22.1, J17.0*A37.9, J17.0*A42.0, J17.0*A43.0, J17.0*A54.8 J16|J17[2-8] J18
J10_PNEUMOBACT	Bacterial pneumoniae	X Diseases of the respiratory system (J10_)	14671	286619	J13 J14 J15, J17.0*, J17.0*A01.0, J17.0*A02.2, J17.0*A21.2, J17.0*A22.1, J17.0*A37.9, J17.0*A42.0, J17.0*A43.0, J17.0*A54.8
J10_BRONCHITIS	Acute bronchitis	X Diseases of the respiratory system (J10_)	13832	323785	J20
J10_BRONCHIOLITIS	Acute bronchiolitis	X Diseases of the respiratory system (J10_)	1754	323785	J21
J10_ACUTELOWERNAS	Unspecified acute lower respiratory infection	X Diseases of the respiratory system (J10_)	4119	323785	J22
J10_CHRONRHINITIS	Chronic rhinitis, nasopharyngitis and pharyngitis	X Diseases of the respiratory system (J10_)	9500	258553	J31
J10_CHRONSINUSITIS	Chronic sinusitis	X Diseases of the respiratory system (J10_)	14639	258553	J32
J10_NASALPOLYP	Nasal polyp	X Diseases of the respiratory system (J10_)	5554	258553	J33
J10_CHRONTONSADEN	Chronic diseases of tonsils and adenoids	X Diseases of the respiratory system (J10_)	38983	258553	J35
J10_PERITONSABSC	Peritonsillar abscess	X Diseases of the respiratory system (J10_)	6670	258553	J36
J10_CHRONLARYNGITIS	Chronic laryngitis and laryngotracheitis	X Diseases of the respiratory system (J10_)	3824	258553	J37
J10_VOCALLARYNX	Diseases of vocal cords and larynx+other diseases of upper respiratory tract, no elsewhere classified	X Diseases of the respiratory system (J10_)	17257	258553	J38-39
J10_BRONCH	Bronchitis, not specified as acute or chronic	X Diseases of the respiratory system (J10_)	2566	283589	J40
J10_SIMPLBRONCH	Simple and mucopurulent chronic bronchitis	X Diseases of the respiratory system (J10_)	1180	283589	J41
J10_BRONCHNAS	Unspecified chronic bronchitis	X Diseases of the respiratory system (J10_)	1035	283589	J42
J10_EMPHYSEMA	Emphysema	X Diseases of the respiratory system (J10_)	1655	283589	J43
J10_COPD	COPD	X Diseases of the respiratory system (J10_)	16410	283589	J43-44
J10_ASTHMA_EXMORE	Asthma (more control exclusions)	X Diseases of the respiratory system (J10_)	37253	187112	J45-46
J10_BRONCHIECTASIS	Bronchiectasis	X Diseases of the respiratory system (J10_)	1967	283589	J47
J10_EXTERLUNG	Lung diseases due to external agents	X Diseases of the respiratory system (J10_)	3863	338636	J60-J70
J10_NECROTIC	Suppurative and necrotic conditions of lower respiratory tract	X Diseases of the respiratory system (J10_)	1290	341209	J85-J86
J10_PLEUREFFUSION	Pleural effusion	X Diseases of the respiratory system (J10_)	3994	328786	J90-91*
J10_PLEURPLAGUE	Pleural plaque	X Diseases of the respiratory system (J10_)	2530	328786	J92
J10_PNEUMOTHORAX	Pneumothorax	X Diseases of the respiratory system (J10_)	1780	328786	J93
J10_PLEUROTH	Other pleural conditions	X Diseases of the respiratory system (J10_)	2648	328786	J94
J10_RESPOTHER	Other diseases of the respiratory system	X Diseases of the respiratory system (J10_)	5660	336839	J95-J99
C3_LUNG_NONSMALL_EXALLC	Non-small cell lung cancer (controls excluding all cancers)	II Neoplasms, from cancer register (ICD-O-3)	3865	259583	C34.[123457]

ICD, International Classification of Diseases; IEU, the MRC Integrative Epidemiology Unit (IEU) at the University of Bristol. More information on respiratory diseases can be found at FinnGen biobank (https://r8.risteys.finngen.fi/). The GWAS summary-level data for the body mass index comes from a published Meta-analysis article (PMID:30124842), and Waist circumference's GWAS summary-level data comes from UK biobank. Both of them were extracted by the IEU Open GWAS project.

### 2.2 The choice of instrumental variables

MR analyses employed IVs mainly SNPs as mediators to investigate the causality between exposures and outcomes. SNPs related to waist circumference and BMI were derived from the IEU open GWAS project. We looked for SNPs that were firmly correlated with exposures at linkage disequilibrium r2<0.001, the genome-wide significance p<5×10^-8^, and clumping window 10,000 kb. The F statistic was employed to ensure that IVs and exposures had a strong correlation. An F statistic of larger than 10 was often regarded as meeting the criteria for a strong correlation (26).

### 2.3 Statistical analysis

The primary way to measure the relationships between exposures and outcomes was the IVW (inverse variance weighted) method. It has the greatest power to detect causality and assumes that the horizontal pleiotropy is balanced or that all SNPs are valid (27). The most significant method to determine if there are any causal associations in this study is the IVW method. In this study, two methods were employed as supplementary: The Methods of Weighted Median and MR-Egger. Less than or equivalent to 50% of invalid IVs are permitted under the weighted median method (28). Even if all IVs were invalid, the MR-Egger method was still able to produce reliable effect estimates (29). The conclusions will be more reliable if the results of the three MR methods are consistent. The IVW model’s heterogeneity was evaluated employing Cochran’s Q test, and heterogeneity was observed if p<0.05. It should be highlighted that the IVW model’s effectiveness is unaffected by the presence of heterogeneity. Horizontal pleiotropy was identified using the MR-Egger intercepts. The influence of a single SNP on the results of the MR analysis was measured using leave-one-out analysis. The MR-PRESSO method was used to identify potential outliers. If any outliers are found, we will reperform the MR analyses. The TwoSampleMR package (30) for R software (version 4.2.0) was used for all analyses.

## 3 Results

### 3.1 Study profile

In this study, the causality between 35 respiratory diseases and obesity was evaluated. As shown in **Supplementary Table 1**, the range of F statistics for BMI-related IVs is from 72.562 to 73.455, and the range of F statistics for waist circumference-related IVs is from 43.205 to 44.419. We found outliers in only some of the MR analyses, and the causalities identified by the IVW method did not change in most analyses before and after the removal of the outliers, except for the relationship between waist circumference and other upper respiratory tract diseases + vocal cords and larynx diseases (ICD-10: J38-39), where the causality changed from present to absent.

### 3.2 Causality between obesity and respiratory diseases

**Figure 1:**
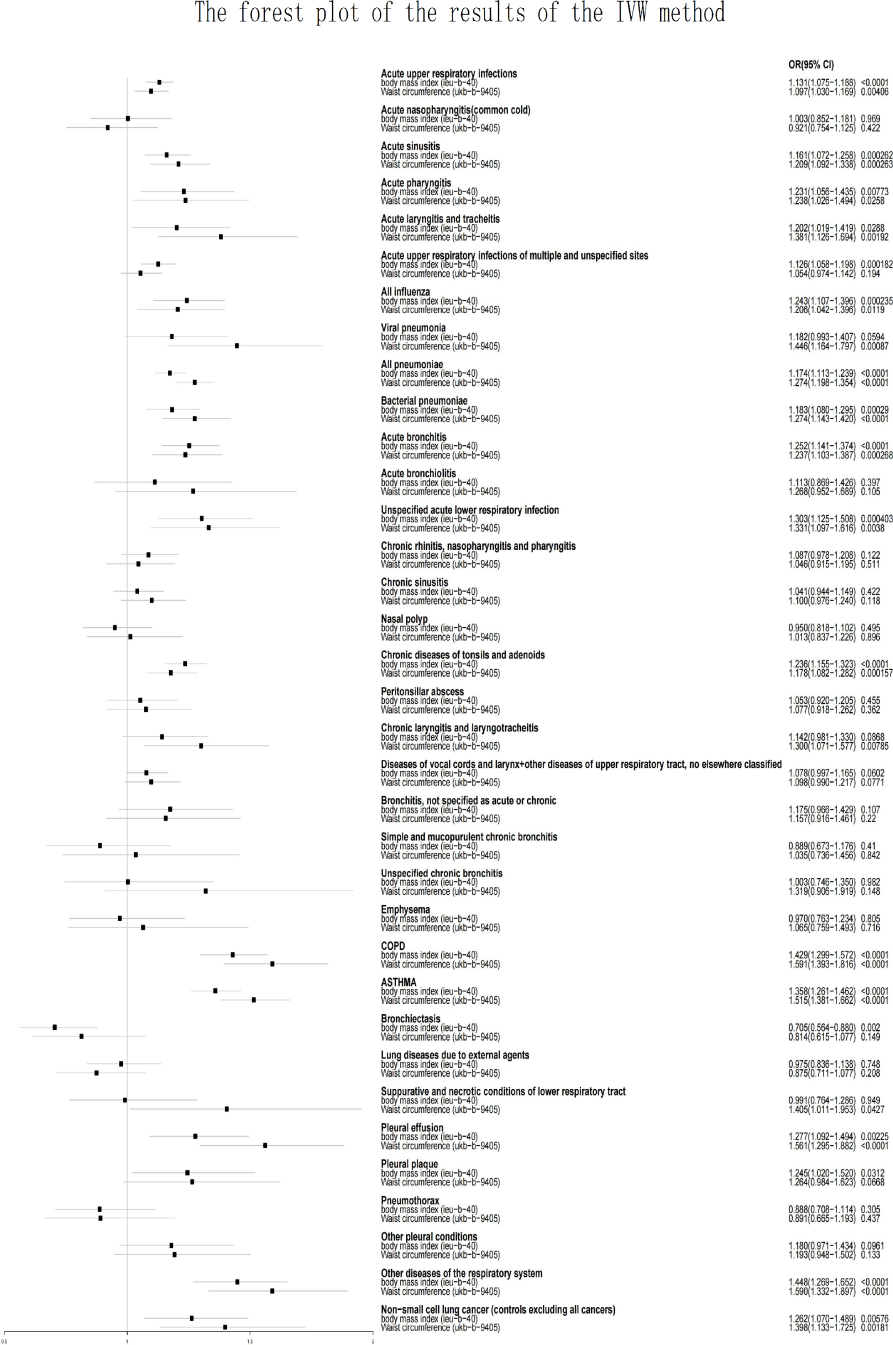
The forest plot of the results of the IVW method.

Horizontal pleiotropy was detected when analyzing whether BMI was associated with acute pharyngitis (p=0.0170) and whether waist Circumference (WC) was associated with mucopurulent and simple chronic bronchitis (p=0.0134) or acute unspecified lower respiratory infection (p=0.00379). The presence of horizontal pleiotropy violates the premise assumption of MR and suggests that IVs are directly linked to outcome (31). Therefore, they will directly be considered invalid analyses. Because both BMI and WC are used as measures of obesity, when one is not valid because of the presence of horizontal pleiotropy, the other will be used as the only indicator to assess whether a causal relationship exists. Acute unspecified lower respiratory infection (BMI: OR=1.303, p=0.000403) and acute pharyngitis (WC: OR=1.238, p=0.0258) were related to obesity. Mucopurulent and simple chronic bronchitis (BMI: OR=0.889, p=0.410) did not show a causal rekationship with obesity. This study demonstrates that obesity increases the risk of the majority of respiratory illnesses, except for bronchiectasis. As shown in **Figure 1** and **Supplementary Table 1**, BMI was associated with a reduced risk of bronchiectasis (OR [odds ratio] =0.705; p=0.00200), whereas WC did not show this effect (OR = 0.814; p=0.149). Acute nasopharyngitis, acute bronchiolitis, unspecified chronic bronchitis, chronic rhinitis, pharyngitis and nasopharyngitis, chronic sinusitis, lung diseases due to external agents, nasal polyp, emphysema, peritonsillar abscess, bronchitis, pneumothorax, larynx and vocal cords diseases + other upper respiratory tract diseases, and other pleural conditions were not related to obesity (including BMI and WC; p>0.05).

Pleural plaque (BMI: OR=1.245, p=0.0312), and acute upper respiratory infections of unspecified and multiple sites (BMI: OR=1.126, p=0.000182) were related to only BMI but not to WC (p>0.05).

Chronic laryngotracheitis and laryngitis (WC: OR=1.300, p=0.00785), viral pneumonia (WC: OR=1.446, p=0.00087), and necrotic and suppurative conditions of lower respiratory tract (WC: OR=1.405, p=0.0427) were related to only WC but not to BMI (p>0.05).

Acute upper respiratory infections (BMI: OR=1.131, p<0.0001; WC: OR=1.097, p=0.00406), acute sinusitis (BMI: OR=1.161, p=0.000262; WC: OR=1.209, p=0.000263), acute tracheitis and laryngitis (BMI: OR=1.202, p=0.0288; WC: OR=1.381, p=0.00192), all influenza (BMI: OR=1.243, p=0.000235; WC: OR=1.206, p=0.0119), all pneumoniae (BMI: OR=1.174, p <0.0001; WC: OR=1.272, p <0.0001), bacterial pneumoniae (BMI: OR=1.183, p=0.000290; WC: OR=1.274, p<0.0001), acute bronchitis (BMI: OR=1.252, p <0.0001; WC: OR=1.237, p=0.000268), chronic tonsils and adenoids diseases (BMI: OR=1.236, p <0.0001; WC: OR=1.178, p=0.000157), COPD (BMI: OR=1.429, p <0.0001; WC: OR=1.591, p <0.0001), asthma (BMI: OR=1.358, p <0.0001; WC: OR=1.515, p <0.0001), pleural effusion (BMI: OR=1.277, p=0.00225; WC: OR=1.561, p<0.0001), other respiratory system diseases (BMI: OR=1.448, p <0.0001; WC: OR=1.590, p <0.0001), NSCLC (BMI: OR=1.262, p=0.00576; WC: OR=1.398, p=0.00181) were related to BMI and WC. More information, including the results of the other two analytical methods and outliers, is provided in **Supplementary Table 1**.

## 4 Discussion

This study revealed that obesity increases the risk of the majority of respiratory diseases (including 20 of all 35 respiratory diseases). This study also discovered that obesity reduces the risk of bronchiectasis and obesity did not show any causality with 14 of the respiratory diseases. In this study, the IVW method was the only way to identify whether a causality existed, and if the other two ways also came to the same result, it would strengthen the conclusion’s persuasiveness. As shown in **Figure 1** and **Supplementary Table 1**, the use of BMI and WC as measures of obesity did not reveal conflicting results in the analysis (e.g. using BMI to demonstrate that obesity increases the risk of certain respiratory diseases, while WC provides the opposite conclusion). There is also a causality between some respiratory diseases and only one exposure factor (BMI or WC), suggesting that body fat distribution may have an impact on the development of these diseases. Due to the presence of horizontal pleiotropy, some of the analyses were invalid. In these cases, we will use a single exposure factor as the basis for determining causality. This study found that obesity has a significantly higher impact on acute respiratory diseases than chronic respiratory diseases; for example, obesity increases the risk of acute sinuses and acute bronchitis but does not increase the risk of chronic sinusitis and chronic bronchitis. Although the mechanisms need to be revealed by further research, this finding is also noteworthy. MR analyses have distinct advantages over observational studies, including the ability to avoid bias brought on by confounding factors and reverse causality. MR falls between interventional epidemiology and observational epidemiology in terms of the hierarchy of evidence (32). However, there have already been some MR studies on the associations between respiratory illnesses and obesity (20–22, 33). There are at least two limitations in their studies. First, in their study, BMI was the only measure used to evaluate obesity. As the most commonly used indicator to evaluate obesity, BMI cannot discriminate between fat mass and lean mass and is unable to identify the differential distribution of fat (34). BMI also cannot distinguish between central and peripheral obesity. Compared with BMI, waist circumference is a better predictor of lung function and a better indicator of metabolic risk (35). Therefore, including waist circumference as a supplement to BMI in the analysis of the relationships between obesity and respiratory diseases would make our study more convincing. The causality between some respiratory diseases and WC alone was also found in this study. Second, there are no systematic MR studies of the relationships between obesity and respiratory diseases. Previous studies only focused on a few respiratory diseases, making it impossible to systematically show how obesity and respiratory diseases are related (20–22, 33, 36, 37). Zhang Z et al. (37) found that body mass index increases the risk of chronic rhinosinusitis. This study showed that both BMI and WC were associated with acute sinusitis but not with chronic sinusitis. A meta-analysis of MR published in 2021 noted that there are few MR studies investigating the effect of body mass index on respiratory disease (20). Their study discovered that BMI was not associated with lung cancer (OR:1.07; 95% CI:0.99-1.15) that having a high BMI increased the risk of developing asthma (OR:1.36; 95% CI: 1.29-1.43) and COPD (OR:1.65; 95% CI: 1.47-1.85). This study showed that both BMI and WC increased the risk of asthma, non-small cell lung cancer and COPD, which is basically consistent with their conclusions. In exploring the relationship between BMI and pneumonia, an MR study found a U-shaped association, with underweight individuals having a significantly higher risk of pneumonia than normal-weight individuals (HR 2.05, 95% CI 1.62–2.59), and obese individuals having a significantly higher risk of pneumonia than normal-weight individuals (HR 1.20, 95% CI 1.11–1.30). Since the data for BMI and WC used in this study are continuous, we were unable to detect this U-shaped association. This is a significant limitation that we hope to overcome in future studies.

We included as many respiratory diseases as possible in this analysis, and a significant proportion of these respiratory diseases were analyzed for the first time using the MR methods to determine their relationships with obesity. This study is therefore extremely important for comprehending the relationship between obesity and common respiratory diseases.

Previous studies have shown that a significant contributor to the development of respiratory illnesses is obesity (38, 39). The mechanism of the link between obesity and respiratory diseases remains to be revealed by more studies, and there are still some studies on its underlying mechanisms. As an active endocrine organ, adipose tissue produces various cytokines and hormones (40, 41). Both mechanical (decreased chest volume) and inflammatory mechanisms may play an important role in the link between obesity and respiratory diseases. Studies have shown that obesity may promote the incidence, severity, and prevalence of asthma, and this process may be reversible (42, 43). The findings of this study need to be carefully considered. The causality detected in this study indicates the impacts of long-term exposure to obesity (BMI and waist circumference). The incidence of respiratory diseases may not be affected by short-term changes in obesity for this reason.

It is important to note some of the strengths of our study. First, BMI and WC were employed as indicators to evaluate obesity, effectively avoiding possible bias caused by different obesity types. Second, the exposure factors were mainly from the UK Biobank, while the respiratory diseases were all from the FinnGen Biobank. Therefore, this study will be able to include a sufficient number of populations, and the population overlap rate between exposures and outcomes will be extremely low. Third, this study included most respiratory diseases in the ICD-10 and systematically assessed the impact of obesity on respiratory diseases. Inevitably, there are limitations to this study. First, we used continuous obesity data (including BMI and waist circumference), so we were unable to identify whether there was a U-shaped association between BMI, waist circumference and respiratory disease (e.g., both higher BMI and lower BMI increased the risk of respiratory disease). Second, our MR analysis was unable to distinguish the role of obesity in respiratory diseases in the populations of different ages and sexes. This is because the data we included did not distinguish between different ages and sexes. Third, because only European populations were included in our study, it is challenging to apply our discoveries to other populations.

## 5 Conclusion

This study revealed that obesity decreases the risk of bronchiectasis and

Obesity raises the risk of pleural plaque, acute upper respiratory infections of unspecified and multiple sites, chronic laryngotracheitis and laryngitis, viral pneumonia, necrotic and suppurative conditions of lower respiratory tract, acute upper respiratory infections, acute sinusitis, acute tracheitis and laryngitis, all influenza, all pneumoniae, bacterial pneumoniae, acute bronchitis, chronic tonsils and adenoids diseases, acute unspecified lower respiratory infection, acute pharyngitis, COPD, NSCLC, asthma, pleural effusion, and other respiratory system diseases.

## Data availability statement

Publicly available datasets were analyzed in this study. This data can be found here: All GWAS data used in this study are available in the IEU open GWAS project (https://gwas.mrcieu.ac.uk/) and FinnGen Biobank (https://r8.risteys.finngen.fi/).

## Ethics statement

Ethical approval was not required for the study involving humans in accordance with the local legislation and institutional requirements. Written informed consent to participate in this study was not required from the participants or the participants’ legal guardians/next of kin in accordance with the national legislation and the institutional requirements. The manuscript presents research on animals that do not require ethical approval for their study.

## Author contributions

The study was designed by WY, MM, and BH. Statistical analyses were performed by WY, YY, YG, JG. The manuscript was written by WY and YY. All authors contributed to the interpretation of data and commented on the manuscript. All authors contributed tog the article and approved the submitted version.
