# Supplementary material for: Obesity and risk for respiratory diseases: a Mendelian randomization study
Source: Front Endocrinol (Lausanne). 2023 Aug 29;14:1197730. doi: 10.3389/fendo.2023.1197730 (PMC10497775; doi:10.3389/fendo.2023.1197730)
Supplement: Supplementary file 1 [file Table_1.docx]

**Supplementary Table 1 The results of Mendelian randomization analyses**

|  | outcome | Exposure | F-statistic | Used SNPs | inverse variance weighted method (Wald ratio) | | Weighted median method | | MR-Egger method | | Cochrane’s Q test | | Pleiotropy | | | MR-PRESSO | | | | | | | Outliers excluded | | | | | | | | | | |
| --- | --- | --- | --- | --- | --- | --- | --- | --- | --- | --- | --- | --- | --- | --- | --- | --- | --- | --- | --- | --- | --- | --- | --- | --- | --- | --- | --- | --- | --- | --- | --- | --- | --- |
|  |  |  |  |  | OR(95% CI) | P-value | OR(95% CI) | P-value | OR(95% CI) | P-value | Q | P-value | MR-Egger intercept | se | P-value | Raw | | | Outliers | outlier-corrected | | | inverse variance weighted method | | Weighted median method | | MR-Egger method | | Cochrane’s Q test | | Pleiotropy | | |
|  |  |  |  |  |  |  |  |  |  |  |  |  |  |  |  | casual estimate | sd | P-value |  | casual estimate | sd | P-value | OR(95% CI) | P-value | OR(95% CI) | P-value | OR(95% CI) | P-value | Q | P-value | MR-Egger intercept | se | P-value |
| **1** | Acute upper respiratory infections | body mass index (ieu-b-40) | 73.147 | 422 | 1.124(1.068-1.184) | <0.0001 | 1.068(0.995-1.146) | 0.0701 | 1.084(0.948-1.239) | 0.239 | 623.733 | <0.0001 | 0.000642 | 0.00111 | 0.564 | 0.117 | 0.0264 | <0.0001 | rs11066188 | 0.123 | 0.0255 | <0.0001 | 1.131(1.075-1.188) | <0.0001 | 1.068(0.994-1.147) | 0.0729 | 1.074(0.943-1.222) | 0.282 | 580.131 | <0.0001 | 0.000910 | 0.00107 | 0.398 |
|  |  | Waist circumference (ukb-b-9405) | 44.215 | 299 | 1.104(1.036-1.177) | 0.00236 | 1.053(0.963-1.153) | 0.259 | 1.060(0.887-1.267) | 0.519 | 432.922 | <0.0001 | 0.000682 | 0.00142 | 0.632 | 0.0992 | 0.0326 | 0.00257 | rs2568958 | 0.0928 | 0.0323 | 0.00435 | 1.097(1.030-1.169) | 0.00406 | 1.052(0.957-1.155) | 0.296 | 1.053(0.883-1.256) | 0.564 | 420.804 | <0.0001 | 0.000688 | 0.00140 | 0.625 |
| **2** | Acute nasopharyngitis(common cold) | body mass index (ieu-b-40) | 73.187 | 421 | 1.003(0.852-1.181) | 0.969 | 0.941(0.713-1.242) | 0.669 | 0.976(0.640-1.490) | 0.912 | 442.854 | 0.213 | 0.000479 | 0.00351 | 0.892 | 0.00319 | 0.0831 | 0.969 | NA | NA | NA | NA | NA | NA | NA | NA | NA | NA | NA | NA | NA | NA | NA |
|  |  | Waist circumference (ukb-b-9405) | 44.279 | 299 | 0.921(0.754-1.125) | 0.422 | 0.780(0.544-1.119) | 0.178 | 0.723(0.414-1.261) | 0.253 | 304.257 | 0.389 | 0.00408 | 0.00444 | 0.360 | -0.0820 | 0.102 | 0.422 | NA | NA | NA | NA | NA | NA | NA | NA | NA | NA | NA | NA | NA | NA | NA |
| **3** | Acute sinusitis | body mass index (ieu-b-40) | 73.248 | 421 | 1.151(1.059-1.250) | 0.000944 | 1.072(0.937-1.227) | 0.308 | 1.149(0.926-1.426) | 0.208 | 569.043 | <0.0001 | 0.0000255 | 0.00179 | 0.989 | 0.140 | 0.0424 | 0.00102 | rs11066188 | 0.150 | 0.0410 | 0.000295 | 1.161(1.072-1.258) | 0.000262 | 1.072(0.940-1.224) | 0.299 | 1.131(0.919-1.393) | 0.247 | 528.064 | 0.000228 | 0.000463 | 0.00173 | 0.789 |
|  |  | Waist circumference (ukb-b-9405) | 44.279 | 299 | 1.209(1.092-1.338) | 0.000263 | 1.129(0.958-1.329) | 0.147 | 1.142(0.860-1.516) | 0.359 | 387.734 | 0.000353 | 0.000955 | 0.00226 | 0.673 | 0.190 | 0.0519 | 0.000310 | NA | NA | NA | NA | NA | NA | NA | NA | NA | NA | NA | NA | NA | NA | NA |
| **4** | Acute pharyngitis | body mass index (ieu-b-40) | 73.087 | 422 | 1.231(1.056-1.435) | 0.00773 | 1.124(0.874-1.444) | 0.362 | 0.789(0.532-1.171) | 0.240 | 456.469 | 0.112 | 0.00784 | 0.00327 | 0.0170 | 0.208 | 0.0781 | 0.00803 | NA | NA | NA | NA | NA | NA | NA | NA | NA | NA | NA | NA | NA | NA | NA |
|  |  | Waist circumference (ukb-b-9405) | 44.279 | 299 | 1.238(1.026-1.494) | 0.0258 | 1.275(0.916-1.774) | 0.149 | 1.181(0.701-1.991) | 0.532 | 311.177 | 0.288 | 0.000789 | 0.00417 | 0.850 | 0.213 | 0.0958 | 0.0265 | NA | NA | NA | NA | NA | NA | NA | NA | NA | NA | NA | NA | NA | NA | NA |
| **5** | Acute laryngitis and tracheitis | body mass index (ieu-b-40) | 73.087 | 422 | 1.202(1.019-1.419) | 0.0288 | 1.088(0.823-1.437) | 0.555 | 1.400(0.912-2.148) | 0.125 | 469.309 | 0.0518 | -0.00268 | 0.00356 | 0.451 | 0.184 | 0.0843 | 0.0293 | NA | NA | NA | NA | NA | NA | NA | NA | NA | NA | NA | NA | NA | NA | NA |
|  |  | Waist circumference (ukb-b-9405) | 44.246 | 300 | 1.381(1.126-1.694) | 0.00192 | 1.290(0.888-1.873) | 0.181 | 1.234(0.699-2.176) | 0.469 | 325.460 | 0.140 | 0.00190 | 0.00454 | 0.676 | 0.323 | 0.104 | 0.00211 | NA | NA | NA | NA | NA | NA | NA | NA | NA | NA | NA | NA | NA | NA | NA |
| **6** | Acute upper respiratory infections of multiple and unspecified sites | body mass index (ieu-b-40) | 73.051 | 423 | 1.120(1.052-1.193) | 0.000424 | 1.037(0.946-1.137) | 0.437 | 1.068(0.907-1.257) | 0.432 | 542.467 | <0.0001 | 0.000845 | 0.00136 | 0.534 | 0.113 | 0.0322 | 0.000470 | rs11066188 | 0.118 | 0.0317 | 0.000208 | 1.126(1.058-1.198) | 0.000182 | 1.038(0.948-1.137) | 0.422 | 1.059(0.902-1.243) | 0.486 | 522.382 | 0.000538 | 0.00108 | 0.00133 | 0.417 |
|  |  | Waist circumference (ukb-b-9405) | 44.308 | 300 | 1.060(0.978-1.149) | 0.157 | 1.012(0.898-1.140) | 0.848 | 0.995(0.795-1.245) | 0.965 | 402.025 | <0.0001 | 0.00106 | 0.00179 | 0.553 | 0.0583 | 0.0412 | 0.158 | rs12001437 | 0.0528 | 0.0406 | 0.195 | 1.054(0.974-1.142) | 0.194 | 1.011(0.894-1.143) | 0.862 | 1.009(0.808-1.259) | 0.936 | 389.688 | 0.000276 | 0.000736 | 0.00177 | 0.678 |
| **7** | All influenza | body mass index (ieu-b-40) | 73.087 | 422 | 1.243(1.107-1.396) | 0.000235 | 1.220(1.014-1.468) | 0.0353 | 1.391(1.029-1.879) | 0.0323 | 479.136 | 0.0261 | -0.00197 | 0.00250 | 0.430 | 0.218 | 0.0592 | 0.000265 | NA | NA | NA | NA | NA | NA | NA | NA | NA | NA | NA | NA | NA | NA | NA |
|  |  | Waist circumference (ukb-b-9405) | 44.279 | 299 | 1.206(1.042-1.396) | 0.0119 | 1.295(1.007-1.665) | 0.0444 | 1.637(1.092-2.456) | 0.0178 | 344.514 | 0.0328 | -0.00512 | 0.00324 | 0.115 | 0.188 | 0.0746 | 0.0124 | NA | NA | NA | NA | NA | NA | NA | NA | NA | NA | NA | NA | NA | NA | NA |
| **8** | Viral pneumonia | body mass index (ieu-b-40) | 73.187 | 421 | 1.182(0.993-1.407) | 0.0594 | 1.154(0.862-1.545) | 0.335 | 1.511(0.963-2.372) | 0.0732 | 386.684 | 0.877 | -0.00434 | 0.00374 | 0.247 | 0.167 | 0.0851 | 0.0501 | NA | NA | NA | NA | NA | NA | NA | NA | NA | NA | NA | NA | NA | NA | NA |
|  |  | Waist circumference (ukb-b-9405) | 44.246 | 300 | 1.446(1.164-1.797) | 0.000870 | 1.072(0.745-1.543) | 0.708 | 1.176(0.643-2.151) | 0.599 | 290.465 | 0.627 | 0.00347 | 0.00482 | 0.472 | 0.369 | 0.109 | 0.000827 | NA | NA | NA | NA | NA | NA | NA | NA | NA | NA | NA | NA | NA | NA | NA |
| **9** | All pneumoniae | body mass index (ieu-b-40) | 73.087 | 422 | 1.174(1.113-1.239) | <0.0001 | 1.163(1.078-1.254) | <0.0001 | 1.153(1.003-1.324) | 0.0458 | 582.536 | <0.0001 | 0.000324 | 0.00115 | 0.779 | 0.160 | 0.0273 | <0.0001 | NA | NA | NA | NA | NA | NA | NA | NA | NA | NA | NA | NA | NA | NA | NA |
|  |  | Waist circumference (ukb-b-9405) | 44.352 | 299 | 1.267(1.191-1.349) | <0.0001 | 1.224(1.115-1.343) | <0.0001 | 1.233(1.036-1.467) | 0.0187 | 358.149 | 0.00959 | 0.000458 | 0.00139 | 0.742 | 0.237 | 0.0319 | <0.0001 | rs13163306 | 0.242 | 0.0312 | <0.0001 | 1.274(1.198-1.354) | <0.0001 | 1.224(1.114-1.344) | <0.0001 | 1.211(1.021-1.435) | 0.0285 | 341.352 | 0.0389 | 0.000852 | 0.00136 | 0.532 |
| **10** | Bacterial pneumoniae | body mass index (ieu-b-40) | 73.187 | 421 | 1.183(1.080-1.295) | 0.000290 | 1.183(1.032-1.357) | 0.0159 | 1.242(0.981-1.572) | 0.0728 | 541.085 | <0.0001 | -0.000853 | 0.00196 | 0.663 | 0.167 | 0.0464 | 0.000326 | NA | NA | NA | NA | NA | NA | NA | NA | NA | NA | NA | NA | NA | NA | NA |
|  |  | Waist circumference (ukb-b-9405) | 44.279 | 299 | 1.274(1.143-1.420) | <0.0001 | 1.322(1.124-1.554) | 0.000743 | 1.439(1.065-1.946) | 0.0186 | 350.465 | 0.0196 | -0.00204 | 0.00241 | 0.397 | 0.242 | 0.0553 | <0.0001 | NA | NA | NA | NA | NA | NA | NA | NA | NA | NA | NA | NA | NA | NA | NA |
| **11** | Acute bronchitis | body mass index (ieu-b-40) | 73.187 | 421 | 1.252(1.141-1.374) | <0.0001 | 1.155(1.005-1.326) | 0.0417 | 1.069(0.840-1.360) | 0.587 | 551.079 | <0.0001 | 0.00279 | 0.00200 | 0.164 | 0.225 | 0.0475 | <0.0001 | NA | NA | NA | NA | NA | NA | NA | NA | NA | NA | NA | NA | NA | NA | NA |
|  |  | Waist circumference (ukb-b-9405) | 44.279 | 299 | 1.237(1.103-1.387) | 0.000268 | 1.204(1.023-1.415) | 0.0251 | 1.048(0.763-1.441) | 0.771 | 378.352 | 0.00110 | 0.00278 | 0.00254 | 0.275 | 0.213 | 0.0584 | 0.000316 | NA | NA | NA | NA | NA | NA | NA | NA | NA | NA | NA | NA | NA | NA | NA |
| **12** | Acute bronchiolitis | body mass index (ieu-b-40) | 72.991 | 423 | 1.113(0.869-1.426) | 0.397 | 1.423(0.947-2.139) | 0.0899 | 1.177(0.620-2.237) | 0.618 | 493.096 | 0.00953 | -0.000990 | 0.00533 | 0.853 | 0.107 | 0.126 | 0.397 | NA | NA | NA | NA | NA | NA | NA | NA | NA | NA | NA | NA | NA | NA | NA |
|  |  | Waist circumference (ukb-b-9405) | 44.246 | 300 | 1.268(0.952-1.689) | 0.105 | 1.127(0.687-1.849) | 0.636 | 1.834(0.828-4.064) | 0.136 | 295.639 | 0.544 | -0.00619 | 0.00636 | 0.331 | 0.237 | 0.146 | 0.104 | NA | NA | NA | NA | NA | NA | NA | NA | NA | NA | NA | NA | NA | NA | NA |
| **13** | Unspecified acute lower respiratory infection | body mass index (ieu-b-40) | 73.092 | 422 | 1.303(1.125-1.508) | 0.000403 | 1.306(1.006-1.696) | 0.0452 | 1.535(1.050-2.244) | 0.0274 | 387.562 | 0.877 | -0.00290 | 0.00315 | 0.358 | 0.264 | 0.0717 | 0.000256 | NA | NA | NA | NA | NA | NA | NA | NA | NA | NA | NA | NA | NA | NA | NA |
|  |  | Waist circumference (ukb-b-9405) | 44.279 | 299 | 1.331(1.097-1.616) | 0.00380 | 1.663(1.196-2.312) | 0.00250 | 2.790(1.638-4.750) | 0.000192 | 334.196 | 0.0730 | -0.0124 | 0.00425 | 0.00379 | 0.286 | 0.0989 | 0.00408 | NA | NA | NA | NA | NA | NA | NA | NA | NA | NA | NA | NA | NA | NA | NA |
| **14** | Chronic rhinitis, nasopharyngitis and pharyngitis | body mass index (ieu-b-40) | 73.237 | 421 | 1.095(0.984-1.218) | 0.0950 | 1.099(0.918-1.317) | 0.305 | 1.001(0.759-1.320) | 0.995 | 500.399 | 0.00418 | 0.00159 | 0.00230 | 0.490 | 0.0909 | 0.0544 | 0.0957 | rs10192119 | 0.0833 | 0.0539 | 0.123 | 1.087(0.978-1.208) | 0.122 | 1.099(0.920-1.312) | 0.297 | 0.996(0.757-1.310) | 0.977 | 489.160 | 0.0101 | 0.00154 | 0.00228 | 0.499 |
|  |  | Waist circumference (ukb-b-9405) | 44.246 | 300 | 1.046(0.915-1.195) | 0.511 | 1.102(0.881-1.378) | 0.394 | 1.220(0.842-1.769) | 0.295 | 358.132 | 0.0107 | -0.00259 | 0.00297 | 0.384 | 0.0448 | 0.0682 | 0.512 | NA | NA | NA | NA | NA | NA | NA | NA | NA | NA | NA | NA | NA | NA | NA |
| **15** | Chronic sinusitis | body mass index (ieu-b-40) | 72.997 | 422 | 1.053(0.951-1.166) | 0.323 | 0.969(0.829-1.133) | 0.694 | 1.034(0.793-1.348) | 0.804 | 696.689 | <0.0001 | 0.000316 | 0.00220 | 0.886 | 0.0515 | 0.0522 | 0.324 | rs11066188;rs3814883;rs9783858 | 0.0403 | 0.0502 | 0.422 | 1.041(0.944-1.149) | 0.422 | 0.962(0.828-1.117) | 0.608 | 1.005(0.779-1.297) | 0.969 | 635.997 | <0.0001 | 0.000623 | 0.00212 | 0.769 |
|  |  | Waist circumference (ukb-b-9405) | 43.950 | 300 | 1.126(0.997-1.271) | 0.0562 | 1.083(0.886-1.325) | 0.436 | 1.024(0.730-1.437) | 0.890 | 447.749 | <0.0001 | 0.00159 | 0.00270 | 0.558 | 0.118 | 0.062 | 0.0572 | rs3814883 | 0.0953 | 0.0610 | 0.119 | 1.100(0.976-1.240) | 0.118 | 1.082(0.896-1.307) | 0.413 | 0.954(0.684-1.330) | 0.781 | 427.069 | <0.0001 | 0.00238 | 0.00265 | 0.369 |
| **16** | Nasal polyp | body mass index (ieu-b-40) | 72.562 | 421 | 0.929(0.799-1.082) | 0.347 | 0.913(0.732-1.138) | 0.417 | 0.770(0.519-1.142) | 0.194 | 600.565 | <0.0001 | 0.00333 | 0.00327 | 0.310 | -0.0728 | 0.0775 | 0.348 | rs1075901;rs10182181 | -0.0518 | 0.0759 | 0.496 | 0.950(0.818-1.102) | 0.495 | 0.961(0.766-1.207) | 0.735 | 0.874(0.592-1.288) | 0.496 | 566.172 | <0.0001 | 0.00146 | 0.00321 | 0.649 |
|  |  | Waist circumference (ukb-b-9405) | 44.279 | 299 | 1.013(0.837-1.226) | 0.896 | 1.060(0.814-1.380) | 0.666 | 0.963(0.565-1.641) | 0.891 | 432.661 | <0.0001 | 0.000841 | 0.00425 | 0.844 | 0.0127 | 0.0976 | 0.896 | NA | NA | NA | NA | NA | NA | NA | NA | NA | NA | NA | NA | NA | NA | NA |
| **17** | Chronic diseases of tonsils and adenoids | body mass index (ieu-b-40) | 73.455 | 421 | 1.227(1.143-1.318) | <0.0001 | 1.183(1.069-1.309) | 0.00114 | 1.052(0.875-1.264) | 0.589 | 775.353 | <0.0001 | 0.00272 | 0.00153 | 0.0752 | 0.205 | 0.0363 | <0.0001 | rs11066188;rs12888955;rs175165;rs6673081;rs7222349 | 0.212 | 0.0345 | <0.0001 | 1.236(1.155-1.323) | <0.0001 | 1.183(1.076-1.301) | 0.000544 | 1.064(0.893-1.267) | 0.489 | 688.482 | <0.0001 | 0.00266 | 0.00145 | 0.0677 |
|  |  | Waist circumference (ukb-b-9405) | 44.419 | 299 | 1.199(1.099-1.309) | 0.0000485 | 1.185(1.054-1.333) | 0.00464 | 1.062(0.832-1.355) | 0.629 | 535.790 | <0.0001 | 0.00204 | 0.00195 | 0.295 | 0.182 | 0.0447 | <0.0001 | rs1056441;rs1182199;rs2376885 | 0.164 | 0.0433 | 0.000190 | 1.178(1.082-1.282) | 0.000157 | 1.179(1.050-1.326) | 0.00558 | 1.090(0.861-1.380) | 0.472 | 494.251 | <0.0001 | 0.00130 | 0.00189 | 0.492 |
| **18** | Peritonsillar abscess | body mass index (ieu-b-40) | 73.021 | 423 | 1.063(0.927-1.218) | 0.382 | 1.030(0.834-1.271) | 0.786 | 1.037(0.728-1.477) | 0.843 | 582.978 | <0.0001 | 0.000439 | 0.00294 | 0.881 | 0.0608 | 0.0696 | 0.383 | rs7780752 | 0.0514 | 0.0689 | 0.456 | 1.053(0.920-1.205) | 0.455 | 1.028(0.828-1.277) | 0.802 | 1.042(0.734-1.478) | 0.819 | 568.471 | <0.0001 | 0.000186 | 0.00290 | 0.949 |
|  |  | Waist circumference (ukb-b-9405) | 44.336 | 300 | 1.117(0.943-1.323) | 0.200 | 1.065(0.824-1.377) | 0.629 | 1.347(0.841-2.157) | 0.216 | 406.267 | <0.0001 | -0.00314 | 0.00376 | 0.405 | 0.111 | 0.0863 | 0.201 | rs10505836;rs1182199 | 0.0740 | 0.0811 | 0.362 | 1.077(0.918-1.262) | 0.362 | 1.056(0.815-1.367) | 0.681 | 1.362(0.876-2.117) | 0.171 | 354.621 | 0.0121 | -0.00395 | 0.00352 | 0.264 |
| **19** | Chronic laryngitis and laryngotracheitis | body mass index (ieu-b-40) | 73.092 | 422 | 1.142(0.981-1.330) | 0.0868 | 1.119(0.851-1.471) | 0.422 | 1.235(0.833-1.830) | 0.295 | 411.159 | 0.625 | -0.00137 | 0.00327 | 0.675 | 0.133 | 0.767 | 0.0839 | NA | NA | NA | NA | NA | NA | NA | NA | NA | NA | NA | NA | NA | NA | NA |
|  |  | Waist circumference (ukb-b-9405) | 44.246 | 300 | 1.300(1.071-1.577) | 0.00785 | 1.633(1.172-2.275) | 0.00377 | 1.662(0.971-2.845) | 0.0647 | 308.589 | 0.339 | -0.00412 | 0.00429 | 0.337 | 0.262 | 0.0986 | 0.00828 | NA | NA | NA | NA | NA | NA | NA | NA | NA | NA | NA | NA | NA | NA | NA |
| **20** | Diseases of vocal cords and larynx+other diseases of upper respiratory tract, no elsewhere classified | body mass index (ieu-b-40) | 72.991 | 423 | 1.078(0.997-1.165) | 0.0602 | 1.049(0.921-1.195) | 0.471 | 0.962(0.786-1.178) | 0.708 | 478.394 | 0.0298 | 0.00200 | 0.00168 | 0.233 | 0.0750 | 0.0399 | 0.0609 | NA | NA | NA | NA | NA | NA | NA | NA | NA | NA | NA | NA | NA | NA | NA |
|  |  | Waist circumference (ukb-b-9405) | 44.354 | 299 | 1.119(1.006-1.244) | 0.0379 | 1.021(0.860-1.212) | 0.810 | 0.950(0.707-1.275) | 0.732 | 398.697 | <0.0001 | 0.00275 | 0.00236 | 0.243 | 0.112 | 0.0542 | 0.0388 | rs76895963;rs7755574 | 0.0932 | 0.0527 | 0.0782 | 1.098(0.990-1.217) | 0.0771 | 1.020(0.863-1.206) | 0.815 | 0.904(0.678-1.207) | 0.495 | 372.658 | 0.00164 | 0.00325 | 0.00230 | 0.160 |
| **21** | Bronchitis, not specified as acute or chronic | body mass index (ieu-b-40) | 73.187 | 421 | 1.175(0.966-1.429) | 0.107 | 1.288(0.928-1.786) | 0.130 | 1.145(0.688-1.903) | 0.603 | 470.315 | 0.0451 | 0.000460 | 0.00422 | 0.913 | 0.161 | 0.100 | 0.108 | NA | NA | NA | NA | NA | NA | NA | NA | NA | NA | NA | NA | NA | NA | NA |
|  |  | Waist circumference (ukb-b-9405) | 44.279 | 299 | 1.157(0.916-1.461) | 0.220 | 1.374(0.929-2.033) | 0.111 | 1.172(0.612-2.245) | 0.632 | 303.276 | 0.404 | -0.000214 | 0.00519 | 0.967 | 0.146 | 0.119 | 0.221 | NA | NA | NA | NA | NA | NA | NA | NA | NA | NA | NA | NA | NA | NA | NA |
| **22** | Simple and mucopurulent chronic bronchitis | body mass index (ieu-b-40) | 73.092 | 422 | 0.889(0.673-1.176) | 0.410 | 0.805(0.501-1.293) | 0.369 | 0.715(0.347-1.474) | 0.364 | 440.934 | 0.242 | 0.00385 | 0.00601 | 0.521 | -0.117 | 0.142 | 0.411 | NA | NA | NA | NA | NA | NA | NA | NA | NA | NA | NA | NA | NA | NA | NA |
|  |  | Waist circumference (ukb-b-9405) | 44.279 | 299 | 1.035(0.736-1.456) | 0.842 | 0.822(0.468-1.444) | 0.495 | 0.337(0.131-0.870) | 0.0252 | 295.833 | 0.525 | 0.0188 | 0.00757 | 0.0134 | 0.0347 | 0.173 | 0.841 | NA | NA | NA | NA | NA | NA | NA | NA | NA | NA | NA | NA | NA | NA | NA |
| **23** | Unspecified chronic bronchitis | body mass index (ieu-b-40) | 73.087 | 422 | 1.003(0.746-1.350) | 0.982 | 1.304(0.786-2.163) | 0.303 | 1.150(0.534-2.480) | 0.721 | 436.518 | 0.291 | -0.00241 | 0.00638 | 0.705 | 0.00348 | 0.151 | 0.982 | NA | NA | NA | NA | NA | NA | NA | NA | NA | NA | NA | NA | NA | NA | NA |
|  |  | Waist circumference (ukb-b-9405) | 43.205 | 299 | 1.319(0.906-1.919) | 0.148 | 0.931(0.510-1.702) | 0.817 | 1.027(0.362-2.919) | 0.959 | 316.386 | 0.222 | 0.00419 | 0.00834 | 0.616 | 0.276 | 0.191 | 0.149 | NA | NA | NA | NA | NA | NA | NA | NA | NA | NA | NA | NA | NA | NA | NA |
| **24** | Emphysema | body mass index (ieu-b-40) | 73.092 | 422 | 0.970(0.763-1.234) | 0.805 | 0.899(0.615-1.316) | 0.585 | 0.665(0.357-1.241) | 0.201 | 457.229 | 0.108 | 0.00665 | 0.00517 | 0.199 | -0.0303 | 0.123 | 0.806 | NA | NA | NA | NA | NA | NA | NA | NA | NA | NA | NA | NA | NA | NA | NA |
|  |  | Waist circumference (ukb-b-9405) | 44.279 | 299 | 1.065(0.759-1.493) | 0.716 | 0.736(0.462-1.172) | 0.197 | 0.760(0.297-1.945) | 0.567 | 408.318 | <0.0001 | 0.00567 | 0.00751 | 0.451 | 0.0628 | 0.172 | 0.716 | NA | NA | NA | NA | NA | NA | NA | NA | NA | NA | NA | NA | NA | NA | NA |
| **25** | COPD | body mass index (ieu-b-40) | 73.185 | 422 | 1.436(1.304-1.581) | <0.0001 | 1.427(1.245-1.635) | <0.0001 | 1.519(1.183-1.951) | 0.00112 | 642.164 | <0.0001 | -0.000999 | 0.00207 | 0.630 | 0.362 | 0.0492 | <0.0001 | rs17663412 | 0.357 | 0.0486 | <0.0001 | 1.429(1.299-1.572) | <0.0001 | 1.426(1.243-1.637) | <0.0001 | 1.517(1.185-1.943) | 0.00102 | 626.198 | <0.0001 | -0.00106 | 0.00205 | 0.607 |
|  |  | Waist circumference (ukb-b-9405) | 44.310 | 300 | 1.596(1.393-1.829) | <0.0001 | 1.673(1.419-1.973) | <0.0001 | 1.821(1.247-2.659) | 0.00209 | 581.906 | <0.0001 | -0.00221 | 0.00302 | 0.465 | 0.468 | 0.0694 | <0.0001 | rs28366156;rs3212038 | 0.464 | 0.0676 | <0.0001 | 1.591(1.393-1.816) | <0.0001 | 1.673(1.423-1.967) | <0.0001 | 1.874(1.297-2.706) | 0.000918 | 545.246 | <0.0001 | -0.00275 | 0.00294 | 0.350 |
| **26** | ASTHMA | body mass index (ieu-b-40) | 73.128 | 421 | 1.375(1.274-1.485) | <0.0001 | 1.374(1.239-1.523) | <0.0001 | 1.415(1.159-1.727) | 0.000703 | 865.529 | <0.0001 | -0.000501 | 0.00166 | 0.762 | 0.319 | 0.0392 | <0.0001 | rs10733051;rs11951673;rs6985109;rs7084454;rs769449 | 0.306 | 0.0377 | <0.0001 | 1.358(1.261-1.462) | <0.0001 | 1.362(1.234-1.503) | <0.0001 | 1.337(1.105-1.620) | 0.00307 | 781.220 | <0.0001 | 0.000270 | 0.00159 | 0.865 |
|  |  | Waist circumference (ukb-b-9405) | 44.084 | 299 | 1.521(1.381-1.674) | <0.0001 | 1.510(1.333-1.710) | <0.0001 | 1.569(1.201-2.050) | 0.00108 | 614.971 | <0.0001 | -0.000523 | 0.00214 | 0.806 | 0.419 | 0.0490 | <0.0001 | rs11653367;rs17296856;rs429358;rs9568867 | 0.416 | 0.0473 | <0.0001 | 1.515(1.381-1.662) | <0.0001 | 1.507(1.324-1.715) | <0.0001 | 1.478(1.143-1.913) | 0.00317 | 553.720 | <0.0001 | 0.000411 | 0.00205 | 0.841 |
| **27** | Bronchiectasis | body mass index (ieu-b-40) | 72.991 | 423 | 0.705(0.564-0.880) | 0.00200 | 0.560(0.381-0.822) | 0.00306 | 0.668(0.376-1.189) | 0.171 | 463.982 | 0.0774 | 0.000935 | 0.00477 | 0.845 | -0.350 | 0.113 | 0.213 | NA | NA | NA | NA | NA | NA | NA | NA | NA | NA | NA | NA | NA | NA | NA |
|  |  | Waist circumference (ukb-b-9405) | 44.279 | 299 | 0.814(0.615-1.077) | 0.149 | 0.540(0.345-0.844) | 0.00694 | 0.484(0.222-1.053) | 0.0684 | 333.689 | 0.0757 | 0.00872 | 0.00621 | 0.162 | -0.206 | 0.143 | 0.150 | NA | NA | NA | NA | NA | NA | NA | NA | NA | NA | NA | NA | NA | NA | NA |
| **28** | Lung diseases due to external agents | body mass index (ieu-b-40) | 73.087 | 422 | 0.975(0.836-1.138) | 0.748 | 0.993(0.770-1.280) | 0.957 | 0.769(0.517-1.146) | 0.198 | 436.267 | 0.294 | 0.00418 | 0.00331 | 0.207 | -0.0253 | 0.0787 | 0.748 | NA | NA | NA | NA | NA | NA | NA | NA | NA | NA | NA | NA | NA | NA | NA |
|  |  | Waist circumference (ukb-b-9405) | 44.279 | 299 | 0.875(0.711-1.077) | 0.208 | 0.973(0.717-1.321) | 0.861 | 0.786(0.441-1.402) | 0.416 | 359.369 | 0.00851 | 0.00179 | 0.00462 | 0.698 | -0.134 | 0.106 | 0.209 | NA | NA | NA | NA | NA | NA | NA | NA | NA | NA | NA | NA | NA | NA | NA |
| **29** | Suppurative and necrotic conditions of lower respiratory tract | body mass index (ieu-b-40) | 73.087 | 422 | 0.991(0.764-1.286) | 0.949 | 1.100(0.697-1.737) | 0.681 | 0.829(0.422-1.626) | 0.585 | 396.730 | 0.797 | 0.00317 | 0.00560 | 0.572 | -0.00856 | 0.129 | 0.947 | NA | NA | NA | NA | NA | NA | NA | NA | NA | NA | NA | NA | NA | NA | NA |
|  |  | Waist circumference (ukb-b-9405) | 44.279 | 299 | 1.405(1.011-1.953) | 0.0427 | 1.523(0.870-2.665) | 0.141 | 0.824(0.330-2.054) | 0.678 | 304.419 | 0.387 | 0.00896 | 0.00730 | 0.221 | 0.340 | 0.168 | 0.0436 | NA | NA | NA | NA | NA | NA | NA | NA | NA | NA | NA | NA | NA | NA | NA |
| **30** | Pleural effusion | body mass index (ieu-b-40) | 73.087 | 422 | 1.277(1.092-1.494) | 0.00225 | 1.017(0.786-1.317) | 0.898 | 1.041(0.693-1.563) | 0.846 | 463.935 | 0.0728 | 0.00360 | 0.00337 | 0.286 | 0.244 | 0.0800 | 0.00240 | NA | NA | NA | NA | NA | NA | NA | NA | NA | NA | NA | NA | NA | NA | NA |
|  |  | Waist circumference (ukb-b-9405) | 44.246 | 300 | 1.561(1.295-1.882) | <0.0001 | 1.304(0.918-1.852) | 0.138 | 1.309(0.779-2.200) | 0.310 | 291.444 | 0.612 | 0.00295 | 0.00415 | 0.477 | 0.445 | 0.0941 | <0.0001 | NA | NA | NA | NA | NA | NA | NA | NA | NA | NA | NA | NA | NA | NA | NA |
| **31** | Pleural plaque | body mass index (ieu-b-40) | 72.991 | 423 | 1.245(1.020-1.520) | 0.0312 | 1.164(0.835-1.623) | 0.371 | 1.619(0.966-2.713) | 0.0684 | 470.415 | 0.0516 | -0.00462 | 0.00428 | 0.281 | 0.219 | 0.102 | 0.0318 | NA | NA | NA | NA | NA | NA | NA | NA | NA | NA | NA | NA | NA | NA | NA |
|  |  | Waist circumference (ukb-b-9405) | 44.273 | 300 | 1.239(0.962-1.596) | 0.0972 | 1.134(0.739-1.740) | 0.564 | 1.032(0.510-2.088) | 0.931 | 342.770 | 0.0413 | 0.00307 | 0.00563 | 0.586 | 0.214 | 0.129 | 0.0982 | rs28375268 | 0.234 | 0.128 | 0.0678 | 1.264(0.984-1.623) | 0.0668 | 1.137(0.737-1.752) | 0.562 | 1.013(0.505-2.032) | 0.970 | 332.901 | 0.0801 | 0.00370 | 0.00556 | 0.506 |
| **32** | Pneumothorax | body mass index (ieu-b-40) | 73.187 | 421 | 0.888(0.708-1.114) | 0.305 | 0.778(0.528-1.145) | 0.203 | 0.841(0.467-1.513) | 0.562 | 438.482 | 0.257 | 0.000973 | 0.00489 | 0.842 | -0.119 | 0.116 | 0.306 | NA | NA | NA | NA | NA | NA | NA | NA | NA | NA | NA | NA | NA | NA | NA |
|  |  | Waist circumference (ukb-b-9405) | 44.279 | 299 | 0.891(0.665-1.193) | 0.437 | 0.754(0.462-1.229) | 0.257 | 1.376(0.611-3.097) | 0.441 | 330.664 | 0.0936 | -0.00730 | 0.00648 | 0.261 | -0.116 | 0.149 | 0.438 | NA | NA | NA | NA | NA | NA | NA | NA | NA | NA | NA | NA | NA | NA | NA |
| **33** | Other pleural conditions | body mass index (ieu-b-40) | 73.087 | 423 | 1.164(0.955-1.419) | 0.132 | 1.050( 0.778-1.416) | 0.752 | 1.490(0.893-2.486) | 0.128 | 494.151 | 0.00872 | -0.00435 | 0.00425 | 0.306 | 0.152 | 0.101 | 0.133 | rs7117238 | 0.165 | 0.0994 | 0.0968 | 1.180(0.971-1.434) | 0.0961 | 1.051(0.771-1.434) | 0.752 | 1.471(0.888-2.435) | 0.135 | 478.056 | 0.0283 | -0.00388 | 0.00418 | 0.354 |
|  |  | Waist circumference (ukb-b-9405) | 44.279 | 299 | 1.193(0.948-1.502) | 0.133 | 0.967(0.655-1.429) | 0.867 | 1.473(0.776-2.796) | 0.237 | 302.347 | 0.419 | -0.00354 | 0.00512 | 0.490 | 0.177 | 0.117 | 0.134 | NA | NA | NA | NA | NA | NA | NA | NA | NA | NA | NA | NA | NA | NA | NA |
| **34** | Other diseases of the respiratory system | body mass index (ieu-b-40) | 73.087 | 422 | 1.448(1.269-1.652) | <0.0001 | 1.643(1.326-2.036) | <0.0001 | 1.693(1.203-2.382) | 0.00268 | 463.330 | 0.0756 | -0.00276 | 0.00284 | 0.332 | 0.370 | 0.0673 | <0.0001 | NA | NA | NA | NA | NA | NA | NA | NA | NA | NA | NA | NA | NA | NA | NA |
|  |  | Waist circumference (ukb-b-9405) | 44.279 | 299 | 1.590(1.332-1.897) | <0.0001 | 1.819(1.376-2.404) | <0.0001 | 2.169(1.327-3.543) | 0.00218 | 377.330 | 0.00124 | -0.00521 | 0.00392 | 0.185 | 0.464 | 0.0902 | <0.0001 | NA | NA | NA | NA | NA | NA | NA | NA | NA | NA | NA | NA | NA | NA | NA |
| **35** | Non-small cell lung cancer (controls excluding all cancers) | body mass index (ieu-b-40) | 73.040 | 422 | 1.282(1.085-1.515) | 0.00349 | 1.099(0.849-1.422) | 0.474 | 1.452(0.942-2.237) | 0.0921 | 498.099 | 0.00566 | -0.00219 | 0.00359 | 0.543 | 0.249 | 0.0852 | 0.00368 | rs11496125 | 0.233 | 0.0843 | 0.00601 | 1.262(1.070-1.489) | 0.00576 | 1.098(0.846-1.426) | 0.481 | 1.434(0.935-2.199) | 0.0990 | 485.220 | 0.0152 | -0.00226 | 0.00355 | 0.525 |
|  |  | Waist circumference (ukb-b-9405) | 44.295 | 299 | 1.425(1.151-1.764) | 0.00114 | 1.212(0.882-1.666) | 0.237 | 1.898(1.049-3.433) | 0.0349 | 368.504 | 0.00332 | -0.00481 | 0.00473 | 0.310 | 0.354 | 0.109 | 0.00128 | rs12375196 | 0.335 | 0.107 | 0.00199 | 1.398(1.133-1.725) | 0.00181 | 1.211(0.878-1.670) | 0.243 | 1.928(1.076-3.455) | 0.0281 | 356.019 | 0.0106 | -0.00540 | 0.00466 | 0.247 |

The heterogeneity was measured employing the Cochran's Q test. The MR-PRESSO method was employed to identify possible outliers. Once any outliers are detected, we repeat all Mendelian randomization analyses and present the results in the ‘Outliers excluded’ section. If the ‘Outliers excluded’ section shows "N/A", no outliers were found. the inverse-variance-weighted (IVW) method is the primary method for identifying causality.The MR‒ Egger and weighted median methods served as a complement to the IVW method. If their results are consistent with the IVW method, the reliability of the IVW method will be greatly improved. The results of MR-PRESSO are presented in the form of beta values, and there is a conversion relationship between beta values and OR, specifically beta=log(OR).

OR: Odds ratio; SNPs: Single-nucleotide polymorphisms; CI: Confidence interval; NA: Not available;
